# The Effect of Metacognitive Instruction on Problem Solving Skills in Iranian Students of Health Sciences

**DOI:** 10.5539/gjhs.v8n1p150

**Published:** 2015-05-15

**Authors:** Yahya Safari, Habibeh Meskini

**Affiliations:** 1 School of Paramedical Sciences, Kermanshah University of Medical Sciences, Kermanshah, Iran; 2 Islamic Azad University of Kermanshah Branch, Kermanshah, Iran; 3 School of Health, Kermanshah University of Medical Sciences, Kermanshah, Iran

**Keywords:** metacognition, problem solving skills, metacognitive instruction, students

## Abstract

**Background:**

Learning requires application of such processes as planning, supervision, monitoring and reflection that are included in the metacognition. Studies have shown that metacognition is associated with problem solving skills. The current research was conducted to investigate the impact of metacognitive instruction on students’ problem solving skills.

**Methods:**

The study sample included 40 students studying in the second semester at Kermanshah University of Medical Sciences, 2013-2014. They were selected through convenience sampling technique and were randomly assigned into two equal groups of experimental and control. For the experimental group, problem solving skills were taught through metacognitive instruction during ten two-hour sessions and for the control group, problem solving skills were taught via conventional teaching method. The instrument for data collection included problem solving inventory (Heppner, 1988), which was administered before and after instruction. The validity and reliability of the questionnaire had been previously confirmed. The collected data were analyzed by descriptive statistics, mean and standard deviation and the hypotheses were tested by t-test and ANCOVA.

**Results:**

The findings of the posttest showed that the total mean scores of problem solving skills in the experimental and control groups were 151.90 and 101.65, respectively, indicating a significant difference between them (p<0.001). This difference was also reported to be statistically significant between problem solving skills and its components, including problem solving confidence, orientation-avoidance coping style and personal control (p<0.001). No significant difference, however, was found between the students’ mean scores in terms of gender and major.

**Conclusion:**

Since metacognitive instruction has positive effects on students’ problem solving skills and is required to enhance academic achievement, metacognitive strategies are recommended to be taught to the students.

## 1. Introduction

The primary objective of educational institutions is students’ learning, and learning requires using such processes as planning, application of knowledge, monitoring, regulation and reflection (Azevedo, 2009), which are included in the domain of metacognition. The term metacognition was first proposed by Flavel (1979). He considers metacognition as the knowledge or metacognitive processes that involve appraisal, monitoring and control of processes and metacognitive activities (Flavel, 1979).

However, scholars relate metacognition to other constructs like meta-learning, critical thinking and motivation (Schneider & Lockl, 2002). Further, Most of the researchers agree upon three components to define metacognition: declarative metacognitive knowledge, cognitive monitoring and regulation of strategies (Alexander, Johnson, Albano, Freygang, & Scott, 2006). Some others consider metacognitive knowledge, metacognitive experiences and metacognitive control strategies as three major components of metacognition that can facilitate our understanding of it (Buwalda, Bouman, & Van Duijin, 2008). From this perspective, metacognitive knowledge refers to the knowledge about one’s cognition such as the information that people have about the memory performance or cognitive control. Metacognitive experiences include one’s appraisal and judgment of the meaning of mental phenomena, and metacognitive control strategies are the individuals’ responses to how they control their cognitive activities (Wells, 2000). The association between these three components in problem solving may be such that a person has unrealistic information about his cognitive ability (metacognitive knowledge) and this uncertainty may be the result of past metacognitive experiences of the person in getting involved with difficult problems or his failures (metacognitive experiences), which may eventually result in the lack of confidence to cope with problems and how to solve them (metacognitive control strategies).

Metacognition is a key factor for prediction of learning performance in the domain of problem solving (Jacobes & Harskamp, 2012). Heppner (1988) introduced three constructs for the problem solving process, including problem solving confidence (believing in one’s abilities to solve the problem), personal control over emotions and behaviors (believing one can control his emotions and behaviors while solving real problems of life) and orientation-avoidance coping styles (the individual’s tendency or avoidance to solve social problems) (Chan, 2001).

According to Sternberg and Sternberg (2012), the steps to problem solving include: identification of problem, representation of problem, formulation of strategies, organization of information, allocation of resources, supervision and evaluation. Studies have confirmed the role of metacognition in problem solving. Accordingly, more complicated problems require more metacognitive control, and teaching problem solving skills and metacognition can provide the learners with proper opportunities to manage their learning (Havenga et al., 2013). Promoting the students’ academic performance through metacognitive strategies is an issue that has been confirmed by some studies (Safari & Arezy, 2012).

The previous studies carried out on metacognition and problem solving show that metacognitive instruction reinforces the students’ ability to better solve the problems because metacognitive strategies enhance their attempt to solve problems (Mokos & Kafoussi, 2013). Studies have shown that students show different metacognitive behaviors to solve mathematical problems in their written descriptions (Pugalee, 2001). Teaching metacognitive strategies promotes the metacognitive skills of the students with learning disorders (Montague, 1992). Likewise, the students instructed via metacognitive approach indicate different levels of problem solving skills compared to control group (Harandi, Eslami Sharbabaki, Ahmadi, & Darehkordi, 2013; Akturk & Sahin, 2011).

Moreover, Anandaraj, and Ramesh (2014) reported a significant correlation between students’ metacognition and problem solving ability. However, metacognitive instruction is more effective in learning environments where metacognitive support is provided during problem solving process than in the environments where this support is provided at the end of problem solving process (Kapa, 2001). In fact, the knowledge of when and how to use metacognitive strategies plays a pivotal role in the students’ success during problem solving process (Teong, 2003). However, metacognitive instruction can help students monitor their understanding and organize their learning and problem solving processes (Brown, 1982).

Teaching and learning researchers have presented similar programs for the metacognitive approach to teaching problem solving skills. Derry and Hawkes (1993) have highlighted two important metacognitive skills for problem solving, including self-monitoring and planning. Self-monitoring is the ability of a person to self-check during problem solving process and planning refers to the ability of an individual to break the problem into secondary objectives that can be separately solved. The metacognitive approach to problem solving instruction was proposed by Kapa (2001). He presented five steps to problem solving and metacognitive operation, including problem identification, problem representation, problem solving planning, performance planning and assessment. Metacognitive operations required for each stage include a) data collection, encoding and reminding, 2) Analogy, inference, imaginativeness, selective comparison and combination, 3) Integration, conceptualization, heuristic choosing and formulating, 4) controlling and monitoring the performance components of algorithmic mathematical knowledge and appropriate rules, and 5) adjusting and contradicting a few possible solutions or suggesting alternative solutions (Kapa, 2001).

Some other researchers, however, have introduced six components for metacognitive instruction, among which metacognitive strategies are suggested for reinforcing problem solving ability. They have argue that two components of specialized knowledge and metacognitive skills are necessary for problem solving. They consider summary tables, flowcharts and causal diagrams etc. as metacognitive strategies that help learners during problem solving process to reduce the content (Schraw, Crippen, & Hartley, 2006). By integrating metacognition and problem solving, Havenga et al. (2013) presented instructional guidelines for the metacognitive approach to problem solving. This program consists of five stages, including 1) studying the problem, underlining the main points, writing the major essentials, revising and articulating, and planning the problem, 2) demonstrating the solution and monitoring the problem, 3) planning the secondary steps (objective, input, process, output), taking the objective and processes of each part or method into account, reflecting on the solutions and motivation for decision-making, 4) coding the planning in a programming language, assessing the program, correcting the program errors and articulating the actions, and 5) testing the program, reflecting on the program codes and programming semantics. The strategies suggested by Montague (1992) for problem solving according to metacognitive process consist of seven stages: 1) study the problem to understand it, 2) paraphrase the problem (in your own words), 3) visualize the problem, 4) hypothesize (a program to solve the problem), 5) estimate (predict the problem), 6) compute (computational operation) and 7) check (make sure everything is right).

In the studies conducted by Howard, McGee, and Hong (2000), five strategies are presented that self-regulated learners use to solve problems, which include problem representation, knowledge of cognition, subtask monitoring, subtask assessment and objectivity. Some studies carried out on the role of learners’ characteristics in metacognitive instruction have shown no significant difference between the learners’ metacognitive processes at different academic levels (Leutwyler, 2009; Safari & Arezi, 2012). Some other studies have indicated a correlation between metacognition and self-efficacy (Coutinho & Neuman, 2009). Learners with high levels of self-efficacy are more successful in metacognitive approach to problem solving skills. They believe that the content of the program is important and useful; hence, they get more involved in it (Aurah, Cassady, & McConnell, 2014).

Since metacognition is an important and appropriate approach to problem solving, the present study was conducted to examine the effect of metacognitive instruction on students’ problem solving skills.

## 2. Methods

This study was a quasi-experimental research with pretest-posttest design. The study sample included 40 undergraduate students of environmental health, public health and food science and industry that were selected trough convenience sampling and randomly divided into two experimental and control groups. The instrument for data collection was problem solving inventory (Heppner, 1988), which was intended to determine the respondents’ understanding of their problem solving behaviors. Heppner proposed three constructs for the problem solving process, including problem solving confidence, personal control over emotions and behaviors, and orientation-avoidance coping styles.

Various theoretical and experimental findings have been presented about metacognitive variables, especially self-evaluation as an effective variable in problem solving. This questionnaire has 35 items that are designed based on 6-point Likert scale to measure how individuals react to their daily activities. According to the results of factor analysis, this questionnaire has three subscales (problem solving confidence, personal control and orientation-avoidance coping style). To avoid bias in responding, 15 statements are presented with a negative tone (they are inversely scored). The total score of the questionnaire is the sum of all responses. Cronbach’s alpha coefficients for problem solving confidence, orientation-avoidance coping style, personal control and the whole problem solving inventory were 0.85, 0.84, 0.72 and 0.86, respectively.

### 2.1 Content of Training Sessions and Administration Style

Problems solving skills were presented to the experimental group through metacognitive approach, but they were presented to the control group via conventional teaching methods. The content of problem solving skills through metacognitive approach was obtained from the history of metacognitive problem solving programs and was validated through a survey by the experts of psychology and education, as follows:

**Table:** 

Implications for problem solving	Metacognitive components
Thinking deeply, reminding the possible strategies (planning)	1. Have I understood whatever is needed for understanding the problem? 2. Disintegration (breaking the problem into its components) 3. Separation 4. Solving the problem components separately
Strategy accomplishment (control and monitoring)	1. Have I determined the patterns and paths for problem solving? 2. Can I write the possible solutions to get the correct response? 3. Am I pressed for time to solve the problem?
Revision and regulation	1. Am I in a right path? 2. Am I approaching the goal? 3. Do I still use the strategies I have selected? 4. Do I need to review the problem again and use another strategy? 5. Can I solve the problem in another way? 6. Is the response correct?

First session: introduction, presentation of the objectives of training workshop, administration of pretest questionnaire, defining the terms, history, metacognitive principles and framework and its role and significance in learning-teaching process.

Second session: teaching metacognitive strategies, teaching major metacognitive strategies (planning, control and monitoring, and regulation strategies) and association of metacognition with problem solving.

Third session: teaching problem solving approaches, highlighting metacognitive problem solving.

Fourth session: demonstration of problem and how to solve it with a metacognitive approach and articulation of details.

Fifth session: presentation of problem and solving the problem using planning strategies by students along with the teacher’s guidance.

Sixth session: solving the problem through control and monitoring strategies.

Seventh session: solving the problem using self-regulation strategies along with the teacher’s guidance.

Eighth session: doing guided metacognitive problem solving exercises in small groups and presentation by the representative of the group and correction of the errors.

Ninth session: independent metacognitive problem solving exercises along with the teacher’s revision.

Tenth session: continuation of exercises, summarization, administration of posttest, closing and donation of rewards.

### 2.2 Data Analysis

The data obtained from the experiment were analyzed by independent t-test and descriptive statistics. In t-test analysis, the pretest in control and experimental groups was considered as a consistent variable and its results were compared with the results of posttest. All statistical analyses were performed by SPSS-16 software.

## 3. Results

The data about the problem solving components (problem solving confidence, orientation-avoidance coping style and personal control) obtained from the pretest and posttest performed in different groups is presented in Table 1.

**Table 1:** Means, Standard Deviations and Comparisons control and experimental groups by pre and post test

Variable	Groups	Pre		Post	
	
		M(SD)	M(SD)	t	p
**Problem Solving Confidence**	cont.	19.95 (9.39)	45.55(7.40)	13.08	0.001
	Exp.	17.55 (6.81)	65.71 (4.74)	50.76	0.001
**Approach- Avoidance**	cont.	27.95 (10.77)	31.45 (5.74)	0.16	0.88
	Exp.	27.10(10.08)	71.65 (4.74)	43.14	0.001
**Personal control**	cont.	23.95(3.05)	28.50 (8.86)	0.98	0.24
	Exp.	25.50- (5.57)	39.50 (6.57)	3.57	0.001
**Problem solving (total)**	cont.	24.85 (14.52)	101.65(6.50)	7.09	0.001
	Exp.	15.25 (14.69)	151.90 (7.32)	50.38	0.001

As shown in Table 1, there is a significant di fference between the means of problem solving components in both experimental and control groups after controlling the pretest variable. This difference was reported to be significant for problem solving component in the experimental group (t=13.08, p<0.001) and in control group (t=50.76, p<0.001); however, it was not significant in the case of orientation-avoidance and personal control components in control group, but it was significant in the case of experimental group. Moreover, for the whole problem solving skills, the experimental group (t=50.38, p<0.001) indicated a better performance than control group (t=7.09, p<0.001). Hence, it can be concluded that metacognitive instruction could promote problem solving skills along with its components in experimental group.

In addition, the data were analyzed in terms of problem solving components and gender. The findings presented in Table 2 indicate no significant difference between the students’ mean scores and gender (Table 2). Also, the results of ANCOVA showed no statistically significant difference between students’ mean scores in different health-related majors and problem solving skills along with its components (Table 3).

**Table 2:** Means, Standard Deviations and Comparisons Problem Solving skills in female and male

Variable	Gender	Post		

		M(SD)	t	p
**Problem Solving skills**	female		55.75(2.56)	0.87
	male		55.38(2.48)	0.46

**Table 3:** Result of ANCOVA of students’ mean scores of problem solving skills in different health-related majors

Sources of variation	Sum of squire	Mean of squire	Df.	f	p
**Between groups**	5.95	2.98	2	0.43	0.63
**Within groups**	238.65	6.42	37		
**total**	243.60		39		

## 4. Discussion

Numerous studies have been conducted on metacognition as an important concept in educational psychology. Some of these studies have investigated the impact of metacognition on other aspects, including problem solving which has been taken seriously into account by the researchers. The main objective of this study was analyzing the effect of metacognitive instruction on students’ problem solving skills. The results of the study showed a significant effect of metacognitive instruction on promoting problem solving skills, including all three components of problem solving (problem solving confidence, personal control and orientation-avoidance coping style). These findings are in in line with the results of the studies conducted by Ramesh (2014) and Anandaraj and Kapa (2001), in which they reported a significant correlation between metacognition and problem solving ability.

Metacognitive approach to teaching regulates the students’ learning process and problem solving (Brown, 1982) and enhances the learners’ problem solving ability through strengthening their efforts to solve the problem (Mokos & Kafoussi, 2013). It also promotes the problem solving skills (Harandi, Eslami Sharbabaki, Ahmadi, & Darehkordi, 2013; Montague, 1992).

The results of the students’ performance in each problem solving component showed that metacognitive approach to problem solving instruction significantly improved the experimental group’s performance in all three problem solving components, as indicated by the results of the posttest compared with those of the pretest. However, no significant difference was observed between the results of pretest and posttest regarding orientation-avoidance and personal control components in control group, but a better performance was reported for problem solving confidence in posttest than in pretest. On the other hand, the effect of conventional teaching method on problem solving confidence was significant in control group. According to the results of the studies performed by Aurah, Cassady, and McConnel (2014), Jacobsen and Harskamp (2012), Schraw, Crippen, Hartley (2006) and Brown (1982) on the effect of metacognitive instruction on promoting the learners’ problem solving skills, and that the abovementioned components are part of problem solving skills, the difference between the performance of experimental group and control group is not far from expectation, but it is noteworthy in the case of control group where problem solving confidence was significantly affected by conventional teaching method, shown by the results of the posttest.

Problem solving confidence is defined as the belief of a person in one’s ability to solve the problem (Heppner, 1988). It seems that this component, in addition to metacognitive approaches to instruction, is affected by other factors, including self-monitoring and planning (Derry & Hawkes, 1993). Although these components are part of metacognitive components, their spontaneous application is possible because they are formed in students as a result of experience. Furthermore, many other strategies such as problem reformulation, cognitive knowledge, monitoring the subtasks, evaluation of subtasks and objectivity, which were proposed by Howard, McGee, Shia and Hong (2000) as the characteristics of self-regulated learners that are effective in problem solving, can be learned through non-metacognitive exercises. Also, metacognition is associated with other constructs like meta-learning, critical thinking and motivation (Schneider & Lockl, 2002), that are believed to be developed by common instructions.

Finally, the comparison of the pretest and posttest mean scores of the students’ problem solving skills indicated no significant difference between the experimental and control groups in terms of gender and major. Some studies analyzing the role of learners’ characteristics in metacognitive instruction have shown no significant difference between metacognitive processes in different genders and levels (Leutwylwr, 2009; Safari & Arezy, 2012). It seems that these variables do not have any effect on metacognitive process and problem solving.

## 5. Conclusion

The findings of the current research revealed that metacognitive problem solving instruction affected the students’ problem solving skills. Hence, an educational course is recommended to be designed in order to strengthen metacognitive strategies and consequently to enhance problem solving skills in students. Further, metacognitive training workshops are suggested to be held for teachers to develop their understanding of this important component of learning-teaching process.
